# Glycan Profiling by Sequencing to Uncover Multicellular Communication: Launching Glycobiology in Single Cells and Microbiomes

**DOI:** 10.3389/fcell.2022.919168

**Published:** 2022-05-27

**Authors:** Lalhaba Oinam, Hiroaki Tateno

**Affiliations:** Cellular and Molecular Biotechnology Research Institute, National Institute of Advanced Industrial Science and Technology, Ibaraki, Japan

**Keywords:** glycan, single cell, glycobiology, microbiome, sequencing, multicellular communication, glycan profiling

## Abstract

Glycans are essential building blocks of life that are located at the outermost surface of all cells from mammals to bacteria and even viruses. Cell surface glycans mediate multicellular communication in diverse biological processes and are useful as “surface markers” to identify cells. Various single-cell sequencing technologies have already emerged that enable the high-throughput analysis of omics information, such as transcriptome and genome profiling on a cell-by-cell basis, which has advanced our understanding of complex multicellular interactions. However, there has been no robust technology to analyze the glycome in single cells, mainly because glycans with branched and heterogeneous structures cannot be readily amplified by polymerase chain reactions like nucleic acids. We hypothesized that the generation of lectins conjugated with DNA barcodes (DNA-barcoded lectins) would enable the conversion of glycan information to gene information, which may be amplified and measured using DNA sequencers. This technology will enable the simultaneous analysis of glycan and RNA in single cells. Based on this concept, we developed a technology to analyze glycans and RNA in single cells, which was referred to as scGR-seq. Using scGR-seq, we acquired glycan and gene expression profiles of individual cells constituting heterogeneous cell populations, such as tissues. We further extended Glycan-seq to the profiling of the surface glycans of bacteria and even gut microbiota. Glycan-seq and scGR-seq are new technologies that enable us to elucidate the function of glycans in cell–cell and cell–microorganism communication, which extends glycobiology to the level of single cells and microbiomes.

## Introduction

The human body is composed of 37 trillion cells, which are covered in a dense layer of glycans with a diameter of ∼30 nm (Martinez-Palomo et al., 1969). Cell surface glycans have been determined to play important roles in diverse biological processes, including development (Haltiwanger and Lowe, 2004), pluripotency (Alisson-Silva et al., 2014; Nishihara, 2018), tumorigenesis (Dube and Bertozzi, 2005; Lau and Dennis, 2008), and immune escape (van Kooyk and Rabinovich, 2008). Cell surface glycans are known to vary depending upon the cell type and state, such as the degree of differentiation and tumorigenesis (Tateno et al., 2011; Hasehira et al., 2012; Taniguchi and Kizuka, 2015; Shimomura et al., 2018). Therefore, glycans are often referred to as a “cell signature” that reflects cellular characteristics (Naoki et al., 2013; Krištić et al., 2014). Most glycans are attached to proteins or lipids to form glycoconjugates, such as glycoproteins, glycolipids, proteoglycans, and glycosylphosphatidylinositol (GPI)-anchored proteins (Gahmberg and Tolvanen, 1996; Kinoshita et al., 1997; Maccioni et al., 2002). Glycans are composed of approximately ten different monosaccharides (Glc, GlcNAc, Gal, GalNAc, Man, Xyl, GlcA, Fuc, NeuAc, and IdoA), which are linked by glycosidic bonds (Cummings and Pierce, 2014; Seeberger, 2015). Anomers and linkage isomers exist in the glycan structure; thus a variety of isomers are possible (Laine, 1994; Werz et al., 2007). Unlike genes and proteins existing as a linear sequence, glycans typically exhibit a branched structure, which is regulated by the substrate specificity of the glycosyltransferases (Varki, 2017). Glycan structures are evolving rapidly, and they are different depending on the organismal species (Varki, 2006; Varki, 2017). Glycans are the secondary products of genes, which are synthesized by the activity of various glycogenes (as of a total >200), such as glycosyltransferases, glycosidases, sugar-nucleotide transporter synthases, and sugar-nucleotide transporters (Yarema and Bertozzi, 2001). As a result, glycan structures may be influenced by intrinsic and extrinsic environmental changes and cannot be easily predicted simply from gene expression profiles. Therefore, it is necessary to develop technologies to analyze cell surface glycans directly.

## Lectin-Based Glycan Profiling

Various strategies have been developed to analyze the glycome, which represents the total set of glycans expressed in a cell or tissue. These techniques include mass spectrometry (MS), high-performance liquid chromatography (HPLC), nuclear magnetic resonance (NMR), and capillary electrophoresis (CE) (Haslam et al., 2006; Yamaguchi and Kato, 2010; Nakano et al., 2011). In 2005, a lectin-based glycan profiling technology, known as lectin microarray, emerged. Since then, it has been noted to play a pivotal role in surveying and mapping the structure of complex glycans in various biological samples (Kuno et al., 2005; Pilobello et al., 2005; Narimatsu et al., 2010; Narimatsu et al., 2018; Hirabayashi et al., 2013; Ribeiro and Mahal, 2013). In lectin microarrays, specific lectins with various glycan-binding specificities, which can discriminate structural isomers such as anomers and linkage isomers of glycans, are immobilized onto glass slides. Glycoproteins extracted from cells or tissues are incubated with the lectin microarray, wherein the binding profiles of lectins can be acquired. Because the specificity of each lectin is known, the glycan profiles and their differences in cells and tissues can be predicted. Lectin microarrays have also been applied to the analysis of not only glycoproteins but also small vesicles, such as exosomes (Gerlach et al., 2013; Shimoda et al., 2017; Shimoda et al., 2022; Saito et al., 2018); viruses (Stevens et al., 2006; Hiono et al., 2019); whole cells, such as live mammalian cells (Zheng et al., 2005; Lee et al., 2006; Tateno et al., 2007; Tao et al., 2008); and bacterial cells (Hsu et al., 2006; Gao et al., 2010; Yasuda et al., 2011). However, there are limitations to lectin microarrays as well as these other analytical methods. For example, 1) glycans cannot be analyzed at the single-cell level, 2) the glycan profile of each cell type in a mixed cell population cannot be obtained without prior separation, and 3) the relationship between the glycome and transcriptome in single cells cannot be determined.

## Concept of Glycan Profiling by Sequencing

**FIGURE 1:**
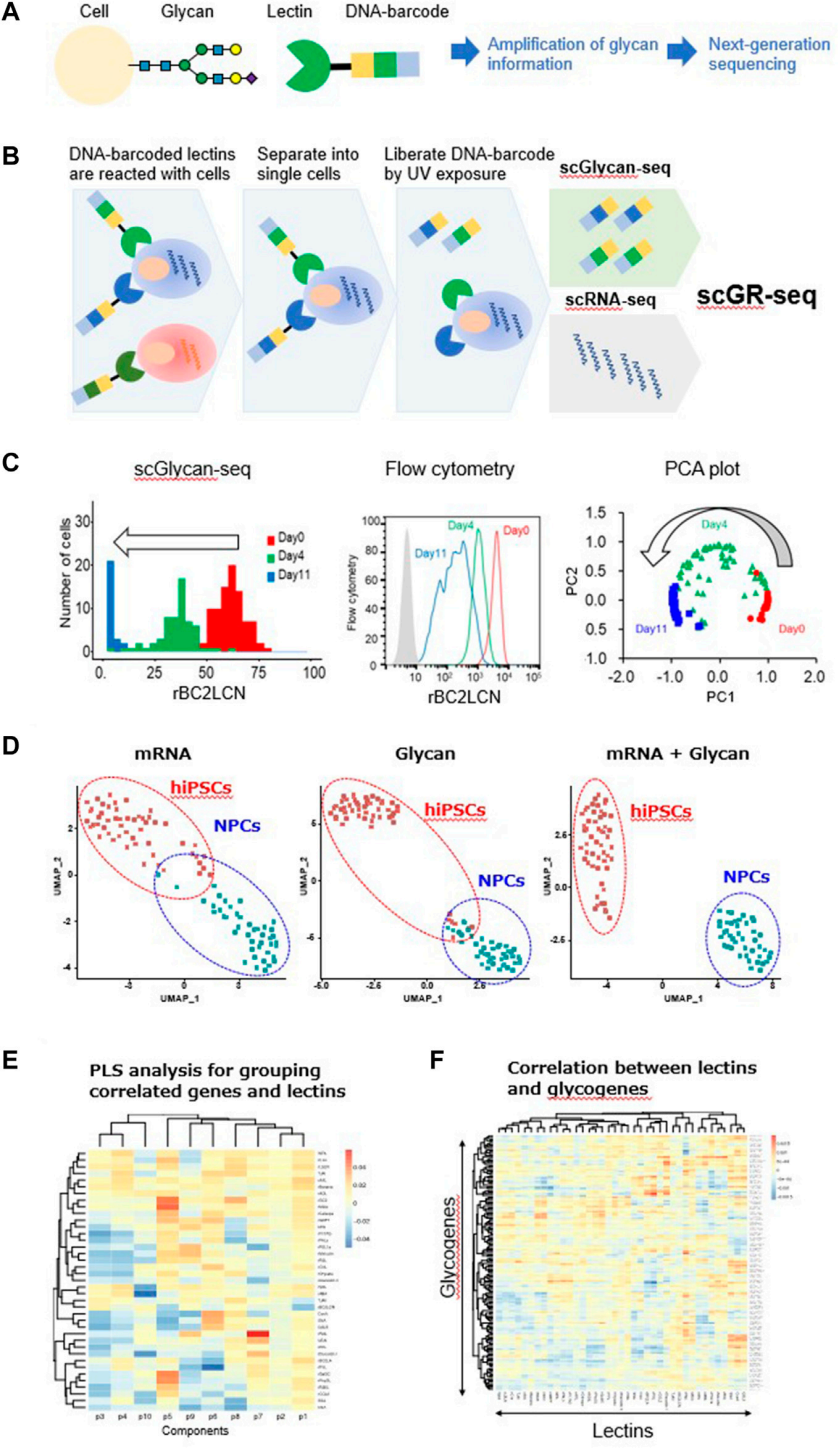
Single-cell glycan and RNA sequencing (scGR-seq). **(A)** Principle of converting glycan information into gene information by DNA-barcoded lectins. **(B)** Schematic experimental workflow of scGR-seq. **(C)** hiPSCs after 0- (*red*), 4- (*green*), and 11-day differentiation (*blue*) into neural progenitor cells were analyzed by scGlycan-seq (left panel), flow cytometry (middle panel), and principal component analysis (right panel). **(D)** Dimensional reduction and clustering. UMAP visualization based on only the scRNA-seq data (left panel), only the scGlycan-seq (middle panel), both scRNA-seq and scGlycan-seq (scGR-seq, right panel) data of hiPSCs (n = 53, *red*), and NPCs (n = 43, *green*). **(E)** PLS regression. A heatmap showing the association between each lectin and each component inferred by PLS regression. Rows represent lectins, and columns represent components. **(F)** Correlation between lectin signal and glycosyltransferase gene expression. A heatmap showing the association of glycogenes and lectins inferred by PLS regression. Rows represent genes, and columns represent lectins. Figures are reprinted from Minoshima et al. (2021) (Minoshima et al., 2021).

High-throughput single-cell sequencing has been transformative for the identification and study of complex cell populations (Stuart and Satija, 2019). Recently, simultaneous profiling of multiple types of molecules within a single cell has been developed in order to establish a more comprehensive molecular view of the cell (Peterson et al., 2017; Stoeckius et al., 2017; Stuart and Satija, 2019). However, there are no techniques yet to simultaneously analyze the glycome and transcriptome in single cells. One reason is that, unlike DNA and RNA, glycans cannot be amplified by methods such as the polymerase chain reaction (PCR). Therefore, we hypothesized that the generation of lectins conjugated with a DNA sequence (DNA-barcoded lectin) would enable the transformation of glycan information to gene information, which may be amplified and further measured using a DNA sequencer, such as a next-generation sequencer (Figure 1A). In addition, the simultaneous analysis of the glycome with other molecular profiles, such as the transcriptome, may be realized.

## Glycan-seq

In 2016, we began developing a technology to analyze glycans in single cells based on the aforementioned concept as a Japan Science and Technology Agency (JST) PRESTO project. Lectins with known specificity were conjugated to DNA oligonucleotides containing a barcode sequence for the identification of the specific lectin, thus enabling the lectins to be specifically identified by sequence analysis (Figure 1A) (Minoshima et al., 2021). The lectins were conjugated *via* their amino groups with the photocleavable dibenzocyclooctyne-N-hydroxysuccinimidyl ester (DBCO-NHS), which allowed the efficient conjugation with 5′-azide-modified oligonucleotides. The oligonucleotides were released from the lectin following ultraviolet (UV) exposure (Odaka et al., 2022). We prepared a panel of 39 DNA-barcoded lectins that covered various glycans, such as sialylated, galactosylated, GlcNAcylated, mannosylated, and fucosylated glycans that are present in glycoconjugates (Minoshima et al., 2021), whereas DNA-barcoded mouse and goat IgG were used as negative controls. In total, 41 DNA-barcoded proteins were incubated with 1 × 10^5^ cells, and the unbound lectins were removed by washing (Odaka et al., 2022) (Figure 1B). Then, the bulk or single cells were separated into a PCR tube and exposed to UV light. After centrifugation, the supernatants containing the released DNA barcodes were recovered, amplified by PCR, and analyzed by a next-generation sequencer to count the DNA barcodes (Odaka et al., 2022). We refer to this method as Glycan-seq.

We evaluated the ability of Glycan-seq in a comparative analysis of the following bulk samples: human-induced pluripotent stem cells (hiPSCs) vs. human dermal fibroblasts (hFibs), Chinese hamster ovary cells vs. glycosylation-defective Chinese hamster ovary mutants, and hiPSCs vs. hiPSC-derived neural progenitor cells (NPCs) (Minoshima et al., 2021). The results were compared by flow cytometry using fluorescence-labeled lectins as the gold standard. Essentially, the Glycan-seq data were consistent with the flow cytometry data (Minoshima et al., 2021). Therefore, bulk Glycan-seq can capture distinct and quantitative differences in glycan profiles in various cell populations as confirmed *via* flow cytometry.

Next, we tested the applicability of Glycan-seq in single cells, which we termed single-cell Glycan-seq (scGlycan-seq) (Minoshima et al., 2021) (Figure 1C). We applied scGlycan-seq for comparative analysis of hiPSCs and hFibs and hiPSCs before and after differentiation into NPCs. The relative quantitative differences in the rBC2LCN signal for hiPSCs before (day 0) and after differentiation to NPCs (days 4 and 11) observed by flow cytometry were also captured by scGlycan-seq (Minoshima et al., 2021) (Figure 1C, left and middle panels). The principal component analysis clearly separated single cells on days 0, 4, and 11, and the cells were clearly ordered with respect to the progression of differentiation (Minoshima et al., 2021) Figure 1C, right panel). Therefore, scGlycan-seq enabled glycan profiling in single cells and revealed cellular heterogeneity in the glycan profiles.

## scGR-seq

scGlycan-seq was then combined with scRNA-seq for the simultaneous analysis of glycan and RNA profiles in single cells (scGR-seq) (Minoshima et al., 2021) (Figure 1B). For scRNA-seq, we used a plate-based method known as RamDA-seq, which is a full-length single-cell total RNA-sequencing method (Hayashi et al., 2018). We performed scGR-seq on human-induced pluripotent stem cells (hiPSCs) and hiPSC-derived NPCs (11-days differentiation). Using UMAP, a nonlinear dimensional clustering based on only the mRNA or glycan data, the two cell types (hiPSCs and NPCs) were partially separated (Minoshima et al., 2021) (Figure 1D). In contrast, when we performed UMAP based on both the mRNA and glycan data, the two cell types were clearly separated (Minoshima et al., 2021) (Figure 1D). Therefore, the combination of mRNA and glycan profiling techniques has further characterized the cell identities. Simultaneous transcriptome and glycome profiles can associate genes with glycans at the single-cell level. A PLS regression analysis was able to identify a group of mRNAs and lectins that were associated with one another differently per component (Minoshima et al., 2021) (Figure 1E). This analysis allowed us to infer each glycan’s potential function and role as a marker through the set of genes associated with the glycan. We also established the overall relationship between lectins and glycosylation-related genes (Minoshima et al., 2021) (Figure 1F). Therefore, scGR-seq is useful for finding potential relevance between the transcriptome and glycome profiles.

## Glycan-seq of the Gut Microbiota

**FIGURE 2:**
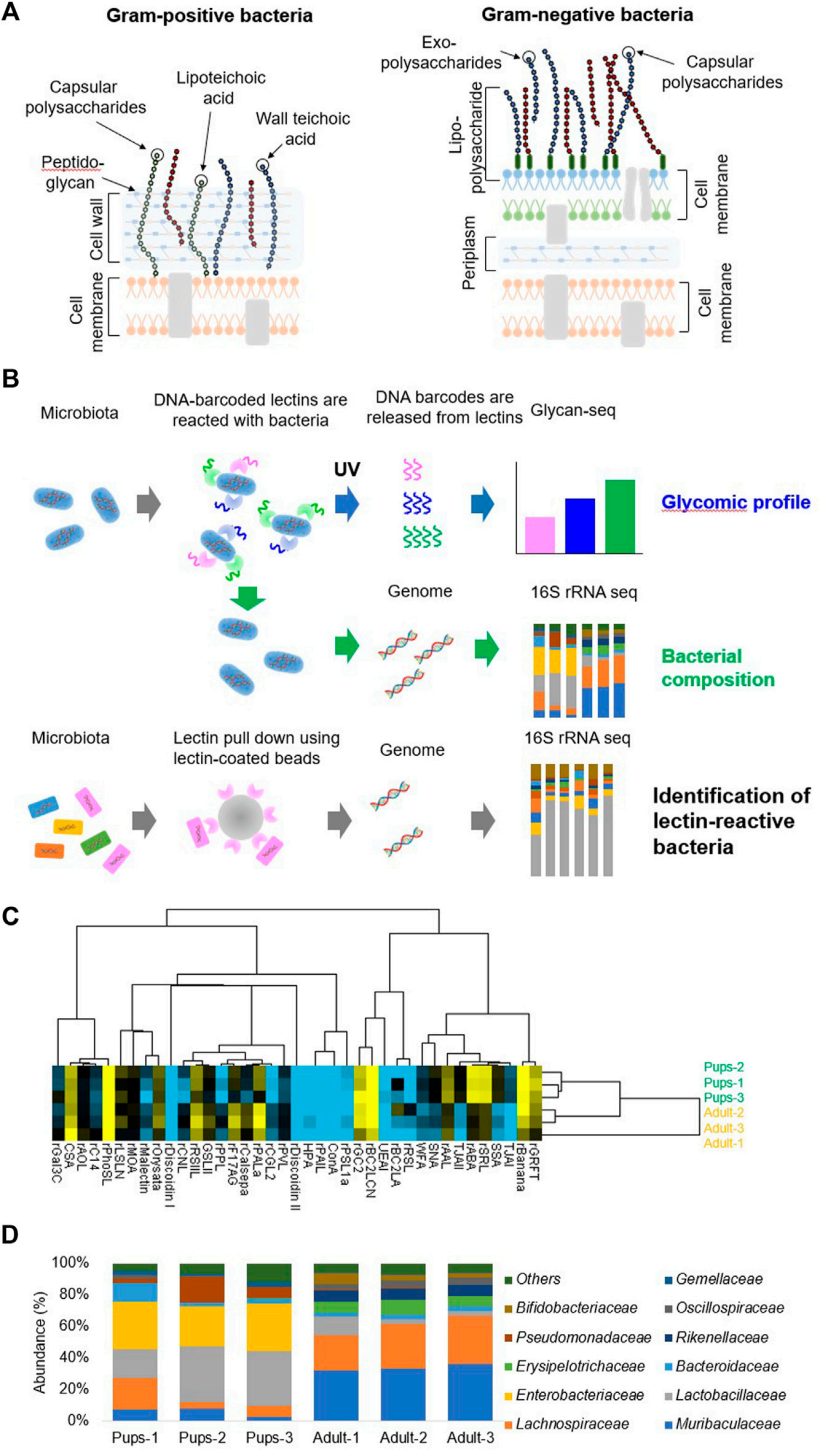
Glycan profiling of the gut microbiota and 16S rRNA sequencing. **(A)** Illustration of the typical cell wall architecture of Gram-positive and negative bacteria. **(B)** Schematic experimental workflow of glycomic profiling, bacterial composition analysis, and the identification of lectin-reactive bacteria. **(C)** Hierarchical clustering heatmap of the gut microbiota of mouse pups (n = 3) and adult mice (n = 3) obtained from the Glycan-seq data. The column shows the pups and adult mouse sample separation, and the row shows the name of the lectins used in the Glycan-seq analysis. **(D)** The stacked bar graph represents the differential abundance of the bacterial family identified by 16S rRNA sequencing from each sample. Each colored bar represents the bacterial family identified. Figures are reprinted from Oinam et al. (2022) (Oinam et al., 2022).

The gut microbiota is known to be populated with diverse microbial communities, of which the bacterial communities are present in high numbers (Costello et al., 2009; Neish, 2009). It is estimated that the human gastrointestinal tract is home to approximately 100 trillion (10^14^) microbes, including 1,000 species of bacteria, which is similar to the number of cells in the entire human body (Sender et al., 2016). The interactions between these microbial communities and the host provide important physiological functions that can affect human health (Sekirov et al., 2010; Kho and Lal, 2018; Valdes et al., 2018; Fan and Pedersen, 2021). Mammalian and bacterial cells are coated with glycans, which serve as an interface for crosstalk with the host (Mescher et al., 1974; Comstock and Kasper, 2006). Bacterial cell surface glycans are highly complex and quite different from those of eukaryotes: Gram-positive bacterial cells are enclosed by a single membrane covered by a thick peptidoglycan layer and lipoteichoic acids (Whitfield, 1988; Rajagopal and Walker, 2017), whereas Gram-negative bacterial cells are covered by two cell membranes (inner and outer membranes) separated by a periplasm containing a thin peptidoglycan layer and the outer membrane consisting of lipopolysaccharides (Figure 2A) (Beveridge, 1999; Casey et al., 2008). Understanding the cell surface glycans of gut bacteria may provide better insight into the interactions between the host and the gut microbiota. Previously, we and others used lectin microarrays to demonstrate that the cell surface glycans of bacteria are different from strain to strain and with culture conditions (Hsu et al., 2006; Gao et al., 2010; Yasuda et al., 2011). Utilizing lectin microarrays for glycan analysis of the gut microbiota is challenging for the following reasons: 1) it is difficult to fluorescently label all of the bacteria comprising the microbiota with the same intensity, 2) a large number of cells is required, and 3) bacterial cells are frequently washed out during the washing steps. Therefore, there has been no robust technology for glycan profiling of the gut microbiota.

We began developing technology for glycan profiling of the gut microbiota as an AMED-Prime project in 2018. For this purpose, the Glycan-seq technique described earlier was modified and implemented for the analysis of bacterial surface glycans. We first evaluated the applicability of Glycan-seq to bacterial glycan profiling in cultured bacteria: Gram-positive (*Deinococcus radiodurans*) vs. Gram-negative (*Escherichia coli*) bacteria (Oinam et al., 2022). After incubation of bacteria with DNA-barcoded lectins, the barcodes were released from the lectins bound to the bacteria by UV irradiation. The recovered DNA barcodes were amplified by PCR, and the number of barcodes derived from each lectin was counted by a next-generation sequencer, which corresponds to the resulting bacterial glycan profiles (Oinam et al., 2022). The Glycan-seq analysis revealed that the glycans were different between Gram-positive and Gram-negative bacteria. To confirm the Glycan-seq data, lectins differentially detected in the cultured bacteria were subjected to flow cytometry analysis, the gold standard for cellular lectin staining. The results indicated that the identified lectins are bound to the bacteria, which is in agreement with the Glycan-seq data and suggested that Glycan-seq data may be applied to bacterial cell surface glycan analysis (Oinam et al., 2022). The specific lectin binding was confirmed using a competition assay with saccharide inhibitors and glycosidase treatment, followed by flow cytometry.

After confirming the applicability of glycan analysis to cultured bacteria, Glycan-seq was used to analyze the gut microbiota along with genomic profiling of the bulk gut microbiota from pups (14–20 days old) and adult (12-month old) mice (Figure 2B) (Oinam et al., 2022). Glycan analysis revealed that the glycans of mouse pups and adult mice were different (Figure 2B) and lectins identifying sialylated glycans were higher in pups (Figure 2C). The genome content was isolated from the bacterial cells, and the V3-V4 region of the 16S rRNA was amplified by PCR (Oinam et al., 2022). The 16S rRNA was then sequenced to determine the bacterial composition of the gut microbiota (Oinam et al., 2022). The composition of the two microbiota was different as bacterial families belonging to Lactobacillaceae, Enterobacteriaceae, Pseudomonadaceae, and Gemellaceae were more abundant in pups (Figure 2D) (Oinam et al., 2022). Sialylated bacteria were then enriched by lectin pulldown using Sia-binding lectins and subjected to 16S rRNA sequencing. The sialylated bacteria identified were Lactobacillaceae, Lachnospiraceae, Enterobacteriaceae, and Muribaculaceae (Oinam et al., 2022). Using Glycan-seq, we were able to perform a glycan analysis of the gut microbiota along with the bacterial composition using the same sequencing instrument. Therefore, Glycan-seq analysis comprehensively revealed differences in the glycan profile of the gut microbiota of pups and adult mice and identified more sialylated bacteria in the mouse pups.

## Concluding Remarks and Future Perspective

scGR-seq provides lectin-based glycan and gene expression profiles for individual cells, making it possible to obtain detailed glycan information and discriminate structural isomers of glycans on single cells constituting a tissue. These data will provide insight into complex multicellular communication networks, including tumor microenvironments and neural networks based on lectin-receptor interactions. scGR-seq can also be applied to the development of drug targets for rare cells, such as cancer stem cells and circulating tumor cells. However, there are limitations to the current Glycan-seq and scGR-seq techniques. Similar to flow cytometry and lectin microarray, absolute amounts of glycans cannot be determined. Another limitation of this current system is the throughput. Since scGR-seq is a plate-based platform, the processing is currently limited to hundreds of cells, whereas it can perform full-length total RNA sequencing. In contrast, droplet-based methods such as 10x Genomics (CITE-seq) can sequence thousands of cells at once but only target the 3′ ends of poly(A) transcripts (Baran-Gale et al., 2018). Because of this difference, scGR-seq will complement the study of single cells in complex biological systems. To resolve this limitation, we plan to improve scGR-seq and adapt it to a droplet-based high-throughput single-cell technology (B Rosenberg et al., 2018). Accordingly, SUrface-protein Glycan and RNA-seq (SUGAR-seq) based on the 10x Genomics platform was also recently reported for the detection of a lectin-binding signal together with the analysis of extracellular epitopes and the transcriptome at the single-cell level (Kearney et al., 2022), although SUGAR-seq detects only one lectin binding to a single cell.

We have also adopted Glycan-seq to approach the untapped glycomics of the gut microbiota, which mediates the direct crosstalk with the host. Bacteria containing a particular glycan surface marker may represent a novel diagnostic and therapeutic target of the disease. For glycomic profiling of the gut microbiota, only bulk analysis by Glycan-seq is currently available. The development of a technique for the simultaneous analysis of the glycome and genome in single cells is needed to fully realize bacterial glycomic profiling in single cells.

In conclusion, we have developed a lectin-based glycan profiling technique by sequencing and applied this technique to the joint analysis of glycan and RNA in single cells and the glycomic profiling of the gut microbiota. Glycan-seq and scGR-seq have the potential to advance our understanding of cellular heterogeneity and the biological role of glycans across diverse multicellular systems across species and lead to the launch of glycobiology in single cells and microbiomes.

## Abbreviations

CE, capillary electrophoresis; HPLC, high-performance liquid chromatography; MS, mass spectrometry; NMR, nuclear magnetic resonance; NPC, neural progenitor cells; and PCR, polymerase chain reaction.
